# Comparison of 2D, 2.5D, and 3D landmark localization networks for 3D cephalometry in CT images

**DOI:** 10.1186/s12903-025-07189-3

**Published:** 2025-11-26

**Authors:** Soo-Min Kim, Min-Hyuk Choi, Seung-Hee Han, Min-Jin Kim, Jo-Eun Kim, Kyung-Hoe Huh, Sam-Sun Lee, Min-Suk Heo, Won-Jin Yi

**Affiliations:** 1 Department of Oral and Maxillofacial Radiology and Dental Research Institute, Seoul National University, Seoul, Korea; 2 Department of Biomedical Radiation Sciences, Graduate School of Convergence Science and Technology, Seoul National University, Seoul, Korea; 3 Department of Orthodontics, School of Dentistry, Kyung Hee University, Seoul, Korea; 4 School of Dentistry, Kyung Hee University, Seoul, Korea

**Keywords:** Deep learning, 3D cephalometry, 2.5D network, Soft voting

## Abstract

**Background:**

Accurate landmark localization is important for three-dimensional (3D) cephalometric analysis. Although deep learning has shown promising performance for 3D landmark localization, the high computational burden of processing volumetric data remains a challenge. The 2.5D networks have emerged to provide the good performance while mitigating computational and memory requirements in the medical domain. Therefore, we compared the performance of 2D, 2.5D and 3D network-based landmark localization.

**Methods:**

We collected landmark datasets from the volumetric computed tomography (CT) scans of 40 patients. We implemented the 2D, 2.5D and 3D networks for 3D landmark localization. Additionally, we designed a global-to-local loss to mitigate foreground-background imbalance, and employed both soft and hard voting in a network ensemble to improve the robustness. We evaluated each network’s performance in terms of accuracy and computational load.

**Results:**

The 2.5D network-based landmark localization achieved a mean radial error (MRE) of 1.19$\:\pm\:$0.65 *mm* and a successful detection rate (SDR) of 86.46% at 2*mm*, with a favorable computational load. These results outperformed those of the 2D and 3D networks. Furthermore, using the global-to-local loss led to higher performance compared to using the global loss alone. Soft voting proved the most robust performance among voting methods for landmark localization.

**Conclusions:**

Comprehensive experiments demonstrate that the 2.5D network offers an optimal trade-off between computational load and accuracy. These findings highlight the potential for more efficient and reliable 3D cephalometry under limited computational resources.

**Supplementary Information:**

The online version contains supplementary material available at 10.1186/s12903-025-07189-3.

## Introduction

Cephalometry, introduced in the 1930 s, has played an important role in dentistry by enabling craniofacial diagnosis, surgical planning, treatment prognosis, and growth analysis [1–6]. Although two-dimensional (2D) cephalometry has been widely adopted over the past few decades [7, 8], its inherent limitation caused by the superimposition of bony structures hinders accurate craniofacial assessment [9–13]. The advent of three-dimensional (3D) imaging systems has overcome this limitation by visualizing anatomical structures without superimposition [6]. Consequently, 3D cephalometry not only enhances the performance of craniofacial analysis but also establishes itself as a superior alternative to conventional 2D cephalometry [14–16].

Landmark localization, which refers to pinpointing pivotal points within the craniofacial structure, is fundamental to cephalometric analysis [6]. Accurate landmark localization is essential for precise analysis [10]. However, manual localization is labor-intensive and time-consuming due to the complexity of craniofacial anatomy and the large volume of 3D data [5]. To address these challenges, automatic methods for 3D landmark localization have been developed [6, 16].

A decade ago, automated landmark localization relied primarily on template matching or statistical shape models [17–21]. Although these methods demonstrated potential to automatic localization, they were susceptible to variability in craniofacial morphology across patients [22]. In recent years, deep learning has emerged as a powerful solution [23–26]. By leveraging diverse patient data with asymmetric craniofacial structures, deep learning-based methods have achieved robust generalization across morphological variations, thereby establishing deep learning as the state-of-the-art methodology for accurate 3D landmark localization [26].

When employing deep learning for landmark localization in computed tomography (CT) images, a critical challenge arises from the massive data size of 3D volumes [16]. This substantial data size significantly increases the demand for computational resources, often exceeding the available hardware capacity and leading to out-of-memory issues [22]. Therefore, memory and computational constraints are key considerations in network design for 3D landmark localization [27]. To mitigate these issues, researchers have explored two strategies [6, 16, 22, 28–30]. The first strategy involves down-sampling volumetric data to reduce the computational load [6, 29]. However, down-sampling results in the loss of fine pixel-level details, thereby compromising the localization accuracy [22]. The second strategy employs a multi-stage approach, wherein first network performs approximate landmark localization on down-sampled volume data, followed by second network operating the accurate localization on the full resolution regions of interests (ROIs) extracted based on initial localization [22, 28, 30, 31]. Although this approach has achieved good performance, it demands substantial resources to implement multiple networks and is susceptible to error propagation and inter-stage dependencies due to separate training [32]. Consequently, there is a growing need for an efficient method that balances memory and computation constraints with localization accuracy.

The network ensemble, referred to as 2.5D networks, has gained prominence as a memory-efficient and accurate solution in 3D medical image applications [33, 34]. This technique involves training 2D networks on axial, sagittal, and coronal image planes and combining their predictions by voting methods for accurate and robust 3D results [35, 36]. Employing 2D networks reduces the computational and memory requirements compared with 3D networks, while ensemble predictions across axial, sagittal, and coronal views enhances robustness over single-plane training [37, 38]. Despite its success in various medical image tasks, its application to 3D landmark localization remains unexplored.

In this study, we compared the landmark localization performance of the 2D, 2.5D, and 3D networks for 3D cephalometry in CT images. Our main contributions are as follows: (1) We conducted a comprehensive quantitative and qualitative comparison of 2D, 2.5D, and 3D networks for landmark localization. (2) We designed the global-to-local loss to accurately identify landmarks under conditions of extreme foreground-background imbalance. (3) We analyzed the effects of soft and hard voting methods within a network ensemble.

## Materials and methods

### Data acquisition and preparation

The CT volumes were obtained from 40 patients via an MDCT scanner (SOMATOM Sensation10, Siemens, Munich, Germany) under 120–140 kVp and 60–80 mAs at Seoul National University Dental Hospital. The patient cohort comprised Koreans aged 12–47 years (21 males and 19 females). Nineteen patients were classified as skeletal class Ⅰ, two as class ⅠⅠ, and nineteen as class ⅠⅠⅠ. The CT volumes were scanned at two resolution types. The first type had dimensions of 512 × 512 pixels with a pixel spacing of 0.35 × 0.35 *mm*^*2*^ and a slice thickness of 0.60 *mm*. The other type had the same dimensions but with 0.58 × 0.58 *mm*^*2*^ and 0.75 *mm*. All volumes were converted to Hounsfield units (HU) using the DICOM Rescale Slope and Intercept, clipped to [−1024, 3071] HU, and normalized to [0, 1]. To standardize the voxel spacing, we cropped to a fixed physical ROI (180 × 180 × 180 *mm*^*3*^) covering the craniofacial region. We then resampled to 512 × 512 × 512 grids with linear interpolation, yielding isotropic voxel spacings of 0.35 × 0.35 × 0.35 *mm*^*3*^. For deep learning, the dataset was randomly divided at the subject (volume) level into 24 for training, 8 for validation, and 8 for testing with fixed seeds. This research was approved by the Institutional Review Board (IRB) of Seoul National University Dental Hospital (ERI18001).

Before designating the landmark locations as the ground truth, all the CT volumes underwent a rotation process to standardize the head orientation, following the method recommended by Cauter et al. [39]. Subsequently, two dentists manually identified the 3D coordinates of landmarks via Amira software (Amira, Thermo Fisher Scientific, Massachusetts, USA). These dentists, who were well-instructed and extensively practiced, ensured precision in landmark localization. The intraclass correlation coefficient (ICC) among the dentist data was 0.999 (*p* < 0.001). The mean of the data was used as the ground truth to guarantee generality. In this study, twelve clinically relevant anatomical landmarks, including the nasion, anterior nasal spine, orbitale (left and right), menton, porion (left and right), sella, point.A, pogonion, and gonion (left and right), were selected [40]. These landmarks are widely used in cephalometry for establishing intracranial references such as the Frankfort horizontal, midsagittal, and sell-nasion planes.

The CT volume was represented as $\:\varvec{I}\in\:{\mathbb{R}}^{\varvec{H}\times\:\varvec{W}\times\:\varvec{D}}$, where $\:\varvec{H}$, $\:\varvec{W}$, and $\:\varvec{D}$ denote the height, width, and depth of volume, respectively. Each landmark was defined as $\:{\varvec{P}}_{\varvec{k}}=\left({\varvec{P}}_{\varvec{k},\varvec{x}},\:{\varvec{P}}_{\varvec{k},\varvec{y}},\:{\varvec{P}}_{\varvec{k},\varvec{z}}\right)$, hear $\:\varvec{k}$ is the landmark index, and $\:{\varvec{P}}_{\varvec{k},\varvec{x}},\:{\varvec{P}}_{\varvec{k},\varvec{y}}$ and $\:{\varvec{P}}_{\varvec{k},\varvec{z}}$ are the voxel coordinates of each landmark within the space of volume $\:\varvec{I}$. We then generated landmark wise 3D heatmaps $\:{\varvec{M}}_{\varvec{k}}\in\:{\mathbb{R}}^{\varvec{H}\times\:\varvec{W}\times\:\varvec{D}},\:$ by applying a Gaussian distribution at each landmark location $\:{\varvec{P}}_{\varvec{k}}$, as described in Eq. (1) [28].

1 $$\:{M}_{k}\left(x,y,z\right)=\frac{1}{\sqrt{2\pi\:}\sigma\:}exp\left(-\frac{{\left(x-{P}_{k,x}\right)}^{2}+{\left(y-{P}_{k,y}\right)}^{2}+{\left(z-{P}_{k,z}\right)}^{2}}{2{\sigma\:}^{2}}\right)$$

where $\:\varvec{x},\:\varvec{y},$ and $\:\varvec{z}$ are the voxel coordinates in each heatmap, and $\:\varvec{\sigma\:}$ denotes the standard deviation, which was empirically set to 8 in this study. In these heatmaps $\:{\varvec{M}}_{\varvec{k}}$, voxels near the landmark exhibited a high value of 1, which gradually decreased to 0 with increasing distance from the landmark. Thus, heatmaps worked as probability maps, indicating the likelihood of the landmark’s location within the CT volume $\:\varvec{I}$.

To generate subsets of data for each direction, we divided the CT volume $$\:\varvec{I}$$ into 2D slice images $$\:{\varvec{i}}_{\varvec{s}}^{\varvec{d}}\::$$$$\:\varvec{d}=\left\{1,\:2,\:3\right\},\:\:\mathbf{s}=\{\text{1,2},\cdots\:,512\}$$ along the axial, sagittal, and coronal directions. Here, $$\:\varvec{d}$$ denotes the axial, sagittal, and coronal directions, and $$\:\varvec{s}$$ is the slice order according to each direction. Therefore, the CT volume $$\:\varvec{I}$$ and slice images $$\:{\varvec{i}}_{\varvec{s}}^{\varvec{d}}$$ are factorized by $$\:\varvec{I}={\left\{{\varvec{i}}_{\varvec{s}}^{\varvec{d}}\right\}}_{\varvec{s}=1}^{512}$$. Similarly, the 3D heatmaps $$\:{\varvec{M}}_{\varvec{k}}$$ were divided into 2D heatmaps $$\:{\varvec{m}}_{\varvec{k},\varvec{s}}^{\varvec{d}}$$ with an equivalent relationship: $$\:{\varvec{M}}_{\varvec{k}}={\left\{{\varvec{m}}_{\varvec{k},\varvec{s}}^{\varvec{d}}\:\right\}}_{\varvec{s}=1}^{512}$$.

All axial, sagittal, and coronal slices for training were obtained exclusively from training subjects, whereas slices for validation and test were solely drawn from their respective held-out subjects. This corresponds to 12,288 slices per direction for training and 4,096 per direction for validation and test.

### 2D network-based landmark localization

In this approach, we first trained a 2D network $\:{\varvec{f}}_{2\varvec{D}}:\:{\varvec{i}}_{\varvec{s}}^{1}\mapsto\:{\varvec{m}}_{\varvec{k},\varvec{s}}^{1}$, which predicted the 2D heatmaps from axial images. After training, we obtained heatmaps $\:{\widehat{\varvec{m}}}_{\varvec{k},\varvec{s}}^{1}$ from the test dataset. These 2D heatmaps $\:{\widehat{\varvec{m}}}_{\varvec{k},\varvec{s}}^{1}$ were then utilized to generate the landmark wise 3D heatmaps $\:{\widehat{\varvec{M}}}_{\varvec{k}}={\left\{{\widehat{\varvec{m}}}_{\varvec{k},\varvec{s}}^{1}\right\}}_{\varvec{s}=1}^{512}$, by stacking them in 3D space according to the slice order $\:\mathbf{s}$. The 3D landmarks $\:{\widehat{\varvec{P}}}_{\varvec{k}}$ were finally identified by the voxel coordinates of the highest value in each 3D heatmap $\:{\widehat{\varvec{M}}}_{\varvec{k}}$ via the following equation: $\:{\widehat{\varvec{P}}}_{\varvec{k}}=\varvec{arg}\varvec{max}\left({\widehat{\varvec{M}}}_{\varvec{k}}\right)$. Consequently, 2D network-based landmark localization was performed via the following equation:

2 $$\:{\widehat{P}}_{k}={arg}{max}\left(\:\:{\left\{\:\:{f}_{2D}\right({i}_{s}^{1}\left)\:\right\}}_{s=1}^{512}\:\:\right)$$

We employed U-Net [41] with an EfficientNet-B3 backbone [42] for the 2D network $\:{\varvec{f}}_{2\varvec{D}}$. The U-Net architecture is composed of an encoder-decoder structure [41]. Despite its simplicity, it has demonstrated powerful performance in the medical image domain [41]. EfficientNet is a convolutional neural network (CNN) designed to balance accuracy and computational efficiency. A common limitation of CNNs is that accuracy improves with model scaling, but this requires considerable computational resources [42]. By using a compound scaling method, EfficientNet-B3 enables higher performance while reducing the computational load [42]. This characteristic is advantageous for landmark localization under constrained resource conditions.

### 2.5D network-based landmark localization

**Fig. 1:**
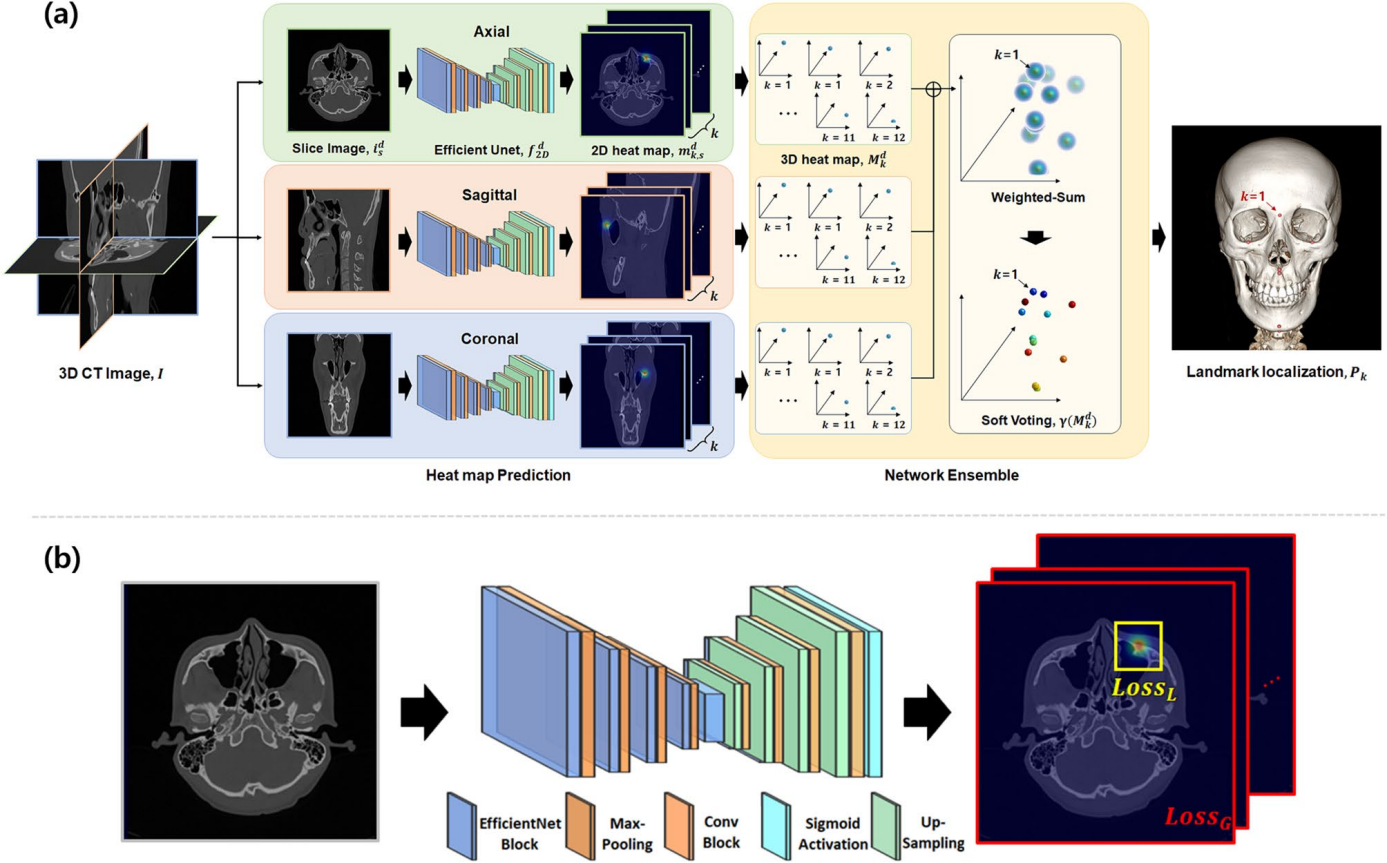
Overview of 2.5D network based landmark localization. **a** Framework for landmark localization from CT volumes. **b** Network architecture for predicting 2D heat map with global-to-local loss

In our 2.5D network-based landmark localization approach, we used the same network architecture described in the previous section. The key difference was that these networks $\:{\varvec{f}}_{2\varvec{D}}^{\:\varvec{d}}:\:{\varvec{i}}_{\varvec{s}}^{\varvec{d}}\mapsto\:{\varvec{m}}_{\varvec{k},\varvec{s}}^{\varvec{d}}$ were independently trained on the axial, sagittal, and coronal datasets, enabling direction-specific learning (Fig. 1(a)). After training, we obtained the predicted 2D heatmaps $\:{\widehat{\varvec{m}}}_{\varvec{k},\varvec{s}}^{\varvec{d}}$ from each test dataset. These 2D heatmaps were subsequently converted into direction-specific 3D heatmaps $\:{\widehat{\varvec{M}}}_{\varvec{k}}^{\varvec{d}}$ by stacking them along each direction. Finally, the 3D landmark location $\:{\widehat{\varvec{P}}}_{\varvec{k}}$ was determined by applying a network ensemble to the direction-specific 3D heatmaps.

In the network ensemble, voting strategies are commonly employed to integrate the results of individual networks [35]. These strategies are categorized into hard and soft voting. Hard voting methods, such as majority, affirmative, and unanimous methods, make binary decisions based on the discrete voting criteria [43, 44]. These methods struggle to fully leverage the continuous probabilistic results by the individual network, which can lead to suboptimal performance. In contrast, soft voting combines the continuous probability from all network results via a weighted summation before the final decision. This approach enhances flexibility, accommodates uncertainty, and improves robustness against misidentification.

We adopted soft voting to combine the direction-specific 3D heatmaps $\:{\widehat{\varvec{M}}}_{\varvec{k}}^{\varvec{d}}$. Soft voting-based landmark localization proceeds through the following steps. First, the weighted sum of 3D heatmaps was computed to integrate the outputs of the individual networks. Next, voxels exceeding a probability threshold of 0.5 were selected from this integrated heatmaps (Eq. 3). Finally, the center of mass (CoM) of these selected voxels was calculated to determine the 3D landmark location (Eq. 4).

3 $$\:\gamma\:\left({\widehat{M}}_{k}^{d}\right)=\left\{\begin{array}{c}1,\:\:\:\:\:\:\:\:\text{i}\text{f}\:\:\:\sum\:_{d}\left({w}^{d}\bullet\:{\widehat{M}}_{k}^{d}\right)\:\:\ge\:0.5\\\:0,\:\:\:\:\:\:\:\text{o}\text{t}\text{h}\text{e}\text{r}\text{w}\text{i}\text{s}\text{e}\:\:\:\:\:\:\:\:\:\:\:\:\:\:\:\:\:\:\:\:\:\:\:\:\end{array}\right.$$

4 $$\:\:{\widehat{P}}_{k}=CoM\left(\:\gamma\:\right({\widehat{M}}_{k}^{d}\left)\:\:\right)$$

Here,$\:\varvec{\gamma\:}$ represents the soft voting functions, $\:{\varvec{w}}^{\varvec{d}}$ denotes each directional weight for soft voting, and $\:\varvec{C}\mathbf{o}\varvec{M}$ refers to the center of mass process applied to the voted voxels. We tuned the weights via a validation-driven grid search, as the equal-weight case [0.33, 0.33, 0.33] and directional dominant cases: [0.50, 0.25, 0.25], [0.25, 0.50, 0.25], and [0.25, 0.25, 0.50]. The final weights were selected by minimizing error on the held-out validation set and then frozen for test evaluation. Consequently, 2.5D network-based landmark localization can be formulated as follows:

5 $$\:{\widehat{P}}_{k}=\:CoM\left(\:\:\gamma\:\right(\:{\left\{\:\:{f}_{2D}^{\:d}\right({i}_{s}^{d}\left)\:\right\}}_{s=1}^{512}\:\left)\:\right)\:\:$$

### 3D Network-based landmark localization

We employed 3D V-Net $\:{\varvec{f}}_{3\varvec{D}}:\varvec{I}\mapsto\:{\varvec{M}}_{\varvec{k}}$ to extract landmark location information from the CT volumes [45]. This network maintained the same configuration as the U-Net, including its skip connections, but replaced the 2D convolutional layers with 3D convolutional layers to process volumetric data. By feeding the 3D volume $\:\varvec{I}$ into the network, we directly obtained the 3D heatmaps $\:{\widehat{\varvec{M}}}_{\varvec{k}}$. Then, the 3D landmarks $\:{\widehat{\varvec{P}}}_{\varvec{k}}$ were identified by locating the voxel coordinates with the maximum value in each 3D heatmap $\:{\widehat{\varvec{M}}}_{\varvec{k}}$, expressed as: $\:{\widehat{\varvec{P}}}_{\varvec{k}}=\varvec{arg}\varvec{max}\left({\widehat{\varvec{M}}}_{\varvec{k}}\right)$. Therefore, 3D network-based landmark localization can be summarized as follows:

6 $$\:{\widehat{P}}_{k}={arg}{max}\left(\:\:{f}_{3D}\right(I\left)\:\:\right)$$

### Global-to-local loss

A pixel wise loss function accumulates the errors across all the pixels in the image during training, which performs adequately when the foreground and background regions are balanced [46–48]. However, in the case of imbalances where the foreground occupies only a small fraction of the entire image, the background dominates the loss. This imbalance reduces the network’s ability to capture foreground features, thereby degrading the overall performance [47]. To overcome this issue, instance wise and region-based losses have been proposed [48–50]. These losses restrict the computational region to balance the foreground and background, ensuring that the background does not dominate the loss.

In this study, the foreground is represented by a small number of heatmaps with a small portion of the images, whereas the background occupies a substantial area [46]. We designed a loss function to capture both image-wide and region-specific features of the heatmaps by employing a combination of global and local loss functions (Fig. 1(b)). The global loss ($\:{\varvec{L}\varvec{o}\varvec{s}\varvec{s}}_{\varvec{G}}$) computes the mean squared error (MSE) between the ground truth and predicted heatmaps across the entire image (Eq. 7), enabling the network to capture the overall feature. Conversely, the local loss ($\:{\varvec{L}\varvec{o}\varvec{s}\varvec{s}}_{\varvec{L}}$) focuses on ROI surrounding each landmark, defined as a 32-pixel area centered on the landmark. By calculating the MSE within the ROI, the local loss highlights fine-grained details (Eq. 8).

7 $$\:{Loss}_G={\parallel\:m_{k,s}^d-\:\widehat m_{k,s}^d\parallel}_2^2$$

8 $$\:{Loss}_L={\parallel\:{\left.m_{k,s}^d\right|}_{ROI}-\:{\left.\widehat m_{k,s}^d\right|}_{ROI}\parallel}_2^2$$

Here, $\:{|}_{\varvec{R}\varvec{O}\varvec{I}}$ denotes the heatmap area within the ROI. For the 3D network, the local term was computed within a cubic ROI centered at each landmark. The total loss ($\:{\varvec{L}\varvec{o}\varvec{s}\varvec{s}}_{\varvec{T}}$) is formulated as the sum of the global and local losses:

9 $$\:{Loss}_{T}={Loss}_{G}+{Loss}_{L}\:$$

This combination allows the network to effectively balance capturing the large-scale features of the entire image and focusing on the detailed features of the foreground, improving localization performance.

### Implementation details

To reduce overfitting, we applied label-preserving augmentation, including rotation within [−10°,10°] and translation up to 5 pixels (or voxels). We intentionally did not use horizontal flips, since mirroring would violate label invariance for bilateral landmarks by swapping left–right anatomy. The network weights were optimized using the Adam optimizer for 150 epochs with a mini-batch size of 8. The learning rate was initially set to 10^− 3^, and was reduced by a factor of 0.5 if the validation loss stagnated for 15 consecutive epochs. Training was conducted on a single GPU (Tesla T4, 16GB, NVIDIA, Santa Clara, CA, USA) via a cloud instance (Google Colab, Google LLC, Mountain View, CA, USA). The networks were implemented via Python 3.11 and PyTorch 2.5.1.

### Performance evaluation

We evaluated the accuracy of landmark localization using complementary metrics. The first metric was the mean radial error (MRE), which quantifies the Euclidean distance between the identified landmark position and the ground truth in millimeters (*mm*) [51]. The second metric was the success detection rate (SDR), which represents the percentage of landmarks with an MRE below predefined thresholds [51, 52]. In this study, we measured the SDR at five thresholds: 1.0 *mm*, 1.5 *mm*, 2.0 *mm*, 2.5 *mm*, and 3.0 *mm*. Among these thresholds, 2.0 *mm* is considered the clinically acceptable criterion [51]. We also reported the floating-point operations (FLOPs), the number of parameters, peak memory (VRAM), and latency per volume for each network to assess computational efficiency and model complexity. The latency was measured from volume to obtain 3D landmarks. These computational metrics are measured under identical hardware.

We conducted several comparative experiments. The main comparison focused on 2D, 2.5D, and 3D networks using various backbones. For 2D and 2.5D networks, we primarily used U-Net with EfficientNet-B3 as the backbone, alongside ResNet18, DenseNet169, and MiT-B2 for benchmarking [53–58]. The MiT-B2 is a transformer-based backbone, whereas the others are the CNN-based backbones. For 3D networks, 3D U-Net and 3D V-Net were used without backbones due to memory constraints [45]. These 3D baselines were trained with patch-based sampling using cubic patches of 64 × 64 × 64 voxels, and matched their training exposure by equalizing the total number of optimizer updates across systems. The second experiment aimed to assess the effectiveness of global-to-local loss in landmark localization. We compared the performance with only the global loss against those using the global-to-local loss. The third experiment compared the voting methods within the network ensemble. We contrasted soft voting with hard voting methods, including majority, affirmative, or unanimous.

To ensure a fair comparison across networks, all hyperparameters and training conditions were kept consistent. Statistical analyses were performed using one-way ANOVA with a significance level of 0.05. Paired analyses used the Wilcoxon signed-rank test with a significance level of 0.05. Confidence intervals for 95% were computed via bootstrap with B = 10,000 resamples (BCa). All analyses were performed via Python’s SciPy library.

## Results

In a validation-set grid search over fusion weights, the coronal-dominant weight [0.25, 0.50, 0.25] yielded the lowest MRE (1.18 mm) and the same configuration achieved numerically best on the test set (1.19 mm) (Supplementary Table 1). Among non-axial-dominant configurations, pairwise differences were not significant, whereas there is a significant difference between the axial-dominant and the others. We therefore adopt a coronal-dominant weight for soft voting in all subsequent analyses.

**Fig. 2:**
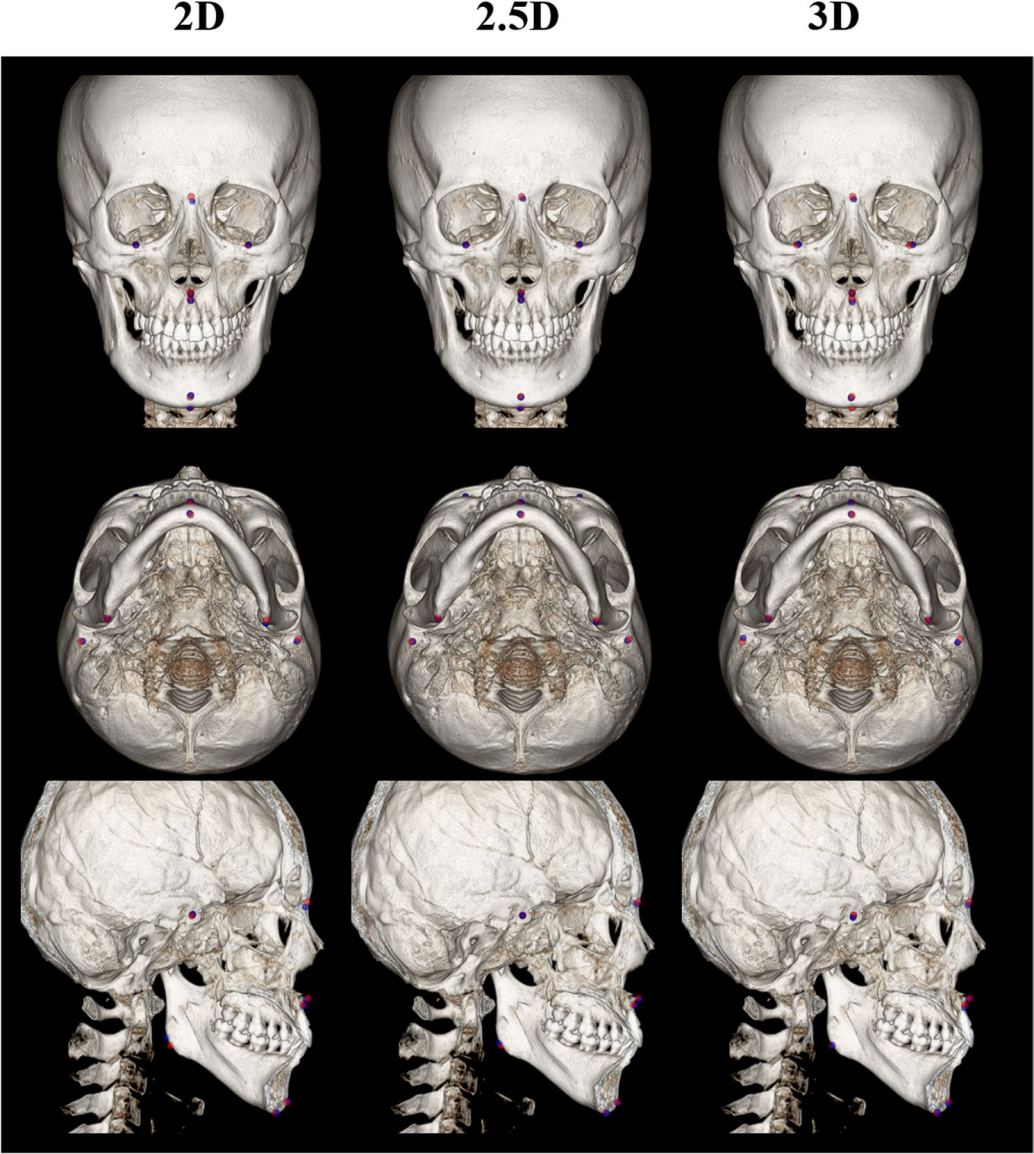
Landmark localization results across dimensional networks and backbone architectures. The 2D and 2.5D network results were acquired using a U-Net with the EfficientNet-B3 backbone, while the 3D results were generated by a V-Net. The red dots indicate ground truth landmarks, whereas the blue dots represent predictions from each network

**Fig. 3:**
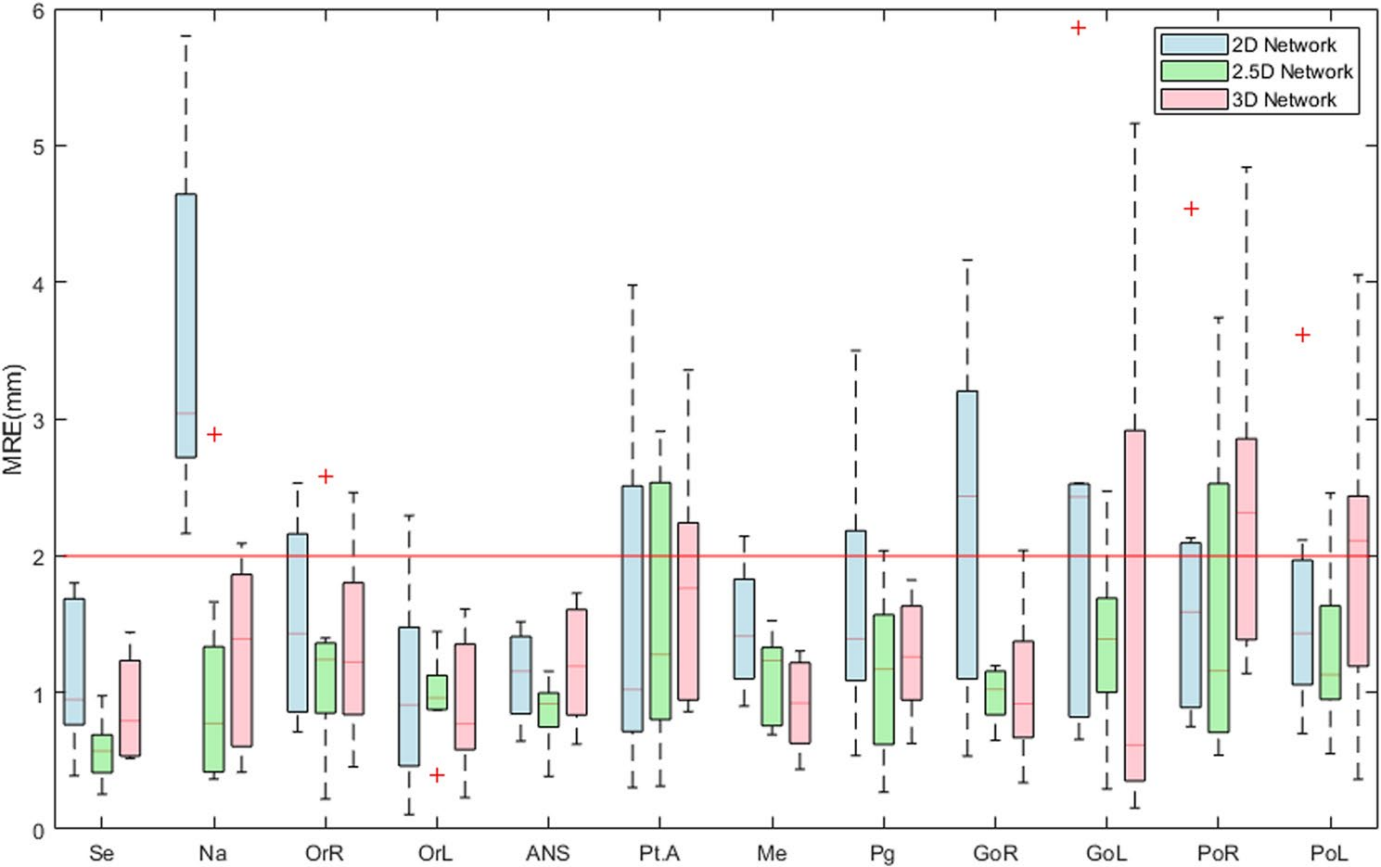
Boxplots of mean radial error (MRE) for each landmark localization across 2D, 2.5D, and 3D networks. The results for 2D and 2.5D networks are obtained from U-Net with EfficientNet-B3 backbone, and the 3D results are obtained from V-Net. The boxes show the median and IQR. Whiskers represent the 5th and 95th percentiles, and the cross indicates outliers. The red line at 2.0 mm denotes the clinical acceptability threshold

Table 1 shows the performance of landmark localization using 2D, 2.5D, and 3D networks across backbones. For the MRE, the 2.5D network with EfficientNet-B3 achieved the lowest value of 1.19 ± 0.65 *mm*, compared with 1.76 ± 1.19 *mm* for the best-performing 2D network and 1.38 ± 0.95 *mm* for the best-performing 3D network. In terms of the SDR, the 2.5D network with EfficientNet-B3 reached 86.46% @ 2.0 *mm*, exceeding the 2D networks, which ranged from 61.46% to 66.67%@ 2.0 *mm*, and the 3D networks, which achieved up to 80.21%@ 2.0 *mm*. In terms of computational efficiency, the 2.5D network with EfficientNet-B3 demonstrated a favorable balance between accuracy and resource consumption. Supplementary Table 2 shows detailed accuracy results, such as 95% CI for the mean, median, and IQR. Pair-wise Wilcoxon signed-rank tests showed that the 2.5D network with EfficientNet-B3 was significantly different from other baselines, except the 2.5D network with DenseNet-169 (*p* = 0.09). Fig. 2 illustrates the landmark localization results across different network dimensions using the best-performing networks in each dimension (2D, 2.5D, and 3D). Fig. 3 presents a comparison of individual landmark localization errors using the best-performing networks in each dimension (2D, 2.5D, and 3D). The detailed accuracy results per landmark are presented in Supplementary Tables 3–4.

**Table 1:** Quantitative evaluation of landmark localization performance across different network dimensions (2D, 2.5D, and 3D) and backbones

Network dimensions	Backbone	Prams ↓ *(M)*	FLOPs↓ *(G)*	Latency↓ *(Sec)*	VRAM↓ *(GB)*	MRE ↓ *(mm)*	SDR ↑ (%)				
							@1.0	@1.5	@2.0	@2.5	@3.0
2 D	**ResNet18**	11.97	17.47	11.84	13.92	1.80$\pm$ 1.20	29.17	46.88	65.63	76.04	87.50
	**DenseNet169**	15.01	33.89	20.32	13.57	1.77$\:\pm\:$1.04	29.17	48.96	65.63	76.04	84.38
	**MiT-B2**	25.04	26.50	20.77	13.99	2.24$\pm$1.82	22.92	42.71	61.46	49.79	76.04
	**EfficientNet-B3**	11.37	11.23	16.92	12.47	176$\:\pm\:$1.19	33.33	52.08	66.67	80.21	86.46
2.5D	**ResNet18**	11.97	17.47	36.69	13.92	1.38$\:\pm\:$0.76	33.33	67.71	81.25	90.63	93.75
	**DenseNet169**	15.01	33.89	60.67	13.57	1.26$\:\pm\:$0.75	41.67	78.13	87.50	91.67	93.75
	**MiT-B2**	25.04	26.50	63.69	13.99	1.39$\:\pm\:$0.89	40.63	71.88	80.21	87.50	91.67
	**EfficientNet-B3**	11.37	11.23	51.52	12.47	1.19$\:\pm\:$0.65	41.67	78.13	86.46	94.79	97.92
3 D	**U-Net**	2.64	14.17	94.80	13.53	1.59$\:\pm\:$0.97	31.25	55.21	75.00	82.29	89.58
	**V-Net**	3.97	24.14	128.57	13.51	1.38$\:\pm\:$0.95	42.71	64.58	80.21	90.63	93.75

Table 2 summarizes the performance of landmark localization via global and local losses across different networks. With the global loss alone, the 2.5D network with EfficientNet-B3 achieved an MRE of 1.48 ± 0.90 *mm*, and an SDR of 77.08% @ 2.0 *mm* threshold. With the global-to-local loss, the MRE decreased to 1.19 ± 0.65 *mm*, and the SDR reached 86.46% @ 2.0 *mm*. For the 2D network, the MRE dropped from 2.32 ± 1.81 *mm* to 1.76 ± 1.19 *mm*. Similarly, the MRE of the 3D network also decreased from 1.42 ± 0.84 *mm* to 1.38 ± 0.95 *mm*. For the 2D and 2.5D networks, the global-to-local loss yielded significantly better performance than the global-only loss (*p* < 0.001). In contrast, for the 3D network, the difference was not significant (*p* = 0.998).

**Table 2:** Landmark localization performance according to the loss function

Network (Backbone)	Loss		MRE ↓ *(mm)*	SDR ↑ (%)				
	Global	Local		@1.0	@1.5	@2.0	@2.5	@3.0
2D U-Net (Efficient-B3)	$\checkmark$		2.32$\:\pm\:$1.81	18.75	36.46	53.13	65.63	72.92
	$\checkmark$	$\checkmark$	1.76$\:\pm\:$1.19	33.33	52.08	66.67	80.21	86.46
2.5D U-Net (Efficient-B3)	$\checkmark$		1.48$\:\pm\:$0.90	37.50	59.38	77.08	85.42	92.71
	$\checkmark$	$\checkmark$	1.19$\:\pm\:$0.65	41.67	78.13	86.46	94.79	97.92
3D V-Net	$\checkmark$		1.42$\:\pm\:$0.84	38.54	64.58	81.25	89.58	93.75
	$\checkmark$	$\checkmark$	1.38$\:\pm\:$0.95	42.71	64.58	80.21	90.63	93.75

Table 3 presents the performance of different voting methods within the ensemble on the 2.5D U-Net with EfficientNet-B3. Soft voting achieved the smallest MRE of 1.19 ± 0.65 *mm* and the highest SDR of 86.46% @ 2.0 *mm*, as well as 78.13% @ 1.5 *mm* and 94.79% @ 2.5 *mm*. Among the hard-voting methods, unanimous voting resulted in the lowest MRE of 1.24 ± 0.72 *mm*, followed by majority voting with an MRE of 1.26 ± 0.63 *mm*. However, the unanimous voting method misidentified six landmarks due to strict voting criteria. Specifically, misidentifications comprised Nasion (*n* = 5) and Porion (*n* = 1). Affirmative voting achieved an MRE of 2.42 ± 1.30 *mm* and an SDR of 46.88% @ 2.0 *mm* threshold, which were the worst performance. In paired comparisons with soft voting, both affirmative (*p* < 0.001) and majority (*p* = 0.008) voting showed significant differences, whereas unanimous voting did not (*p* = 0.090). 

**Table 3:** Landmark localization performances by the voting methods on the 2.5D U-Net with Efficient-B3

	MRE ↓ (mm)	SDR ↑ (%)				
		@1.0	@1.5	@2.0	@2.5	@3.0
Affirmative	2.42$\:\pm\:$1.30	13.54	33.33	46.88	57.29	67.71
Majority	1.26$\:\pm\:$0.63	44.79	76.04	86.46	93.75	98.96
Unanimous	1.24$\:\pm\:$0.72^*^	41.67	71.88	81.25	85.42	91.67
Soft	1.19$\:\pm\:$0.65	41.67	78.13	86.46	94.79	97.92

(*Unanimous misidentified 6 landmarks)

## Discussion

In the field of dentistry, cephalometry plays a key role in diagnosis, surgical planning, and treatment prognosis [1–6]. This technique assesses craniofacial structures by identifying anatomical landmarks [6]. Thus, accurate landmark localization is critical for performing cephalometric analysis correctly [6]. With the widespread adoption of 3D imaging, cephalometry has transitioned to a 3D approach, which enables a more precise assessment of craniofacial anatomy [14–16]. However, the inherent complexity of craniofacial structures and the extensive data associated with 3D images result in manual landmark localization being both time-consuming and labor-intensive [5]. Consequently, there is increasing demand for automatic 3D landmark localization [6].

Initially, template matching and statistical shape models were employed for landmark localization. However, these methods posed a challenge to the generalization of landmark localization due to the variability of craniofacial structures across patients [22]. Recently, deep learning-based approaches have been adopted for automatic landmark localization, demonstrating promising improvements in accuracy [59]. Despite these advancements, the substantial computational resources for training networks with volumetric data have often resulted in out-of-memory issues [22]. To address these issues, several strategies have been proposed to optimize memory usage [27, 59]. First, down-sampling of volumetric data was applied to reduce the computational load during training. However, this approach compromised accuracy due to the increased physical distance between voxel grids [6]. Second, multi-stage frameworks have been proposed [60]. In these frameworks, an initial stage detected approximate regions of interest for landmarks using the down-sampled resolution, and a subsequent stage accurately identified the landmark within that region using the original resolution. Although this approach performed landmark localization on voxel grids at the original resolution, it required separate training for each stage, resulting in susceptibility to inter-stage dependency [22, 28].

In order to optimize the trade-off between performance and computational resource, we employed 2.5D network-based landmark localization using network ensemble. This method reduced the computational burden by training the axial, sagittal, and coronal directional images with multiple 2D networks, and enhanced the performance by integrating the results obtained from the 2D networks. Additionally, we designed the global-to-local loss function to address the imbalance between the foreground and background, and explored more suitable voting schemes for the effective integration of the 2D network results. In this study, we compared the performance and computational efficiency of 2.5D networks with those of conventional 2D and 3D networks for landmark localization.

The performance of 2D, 3D, and 2.5D-based landmark localization were comparatively analyzed in terms of accuracy and computational efficiency (Table 1). In 2D-based landmark localization, networks were trained on each slice independently, which lacked the volumetric context required for accurate 3D localization [31]. This limitation resulted in more false positives, thereby degrading the localization accuracy. Even the best-performing 2D network, EfficientNet-B3, achieved the MRE of 1.76 ± 1.19 *mm* and the SDR of 66.67% @ 2.0 *mm* (Table 1) despite the shortest latency and favorable cost. The 3D network leveraged volumetric context, but required patch-wise training and sliding-window inference due to memory constraints. This led to restricting the field-of-view and increasing latency. The best-performing 3D network, V-Net, achieved an MRE of 1.38 ± 0.95 *mm* and an SDR of 80.21% @ 2.0 *mm* (Table 1). Although 3D networks outperformed 2D networks, they incurred substantially higher computational cost and latency.

**Fig. 4:**
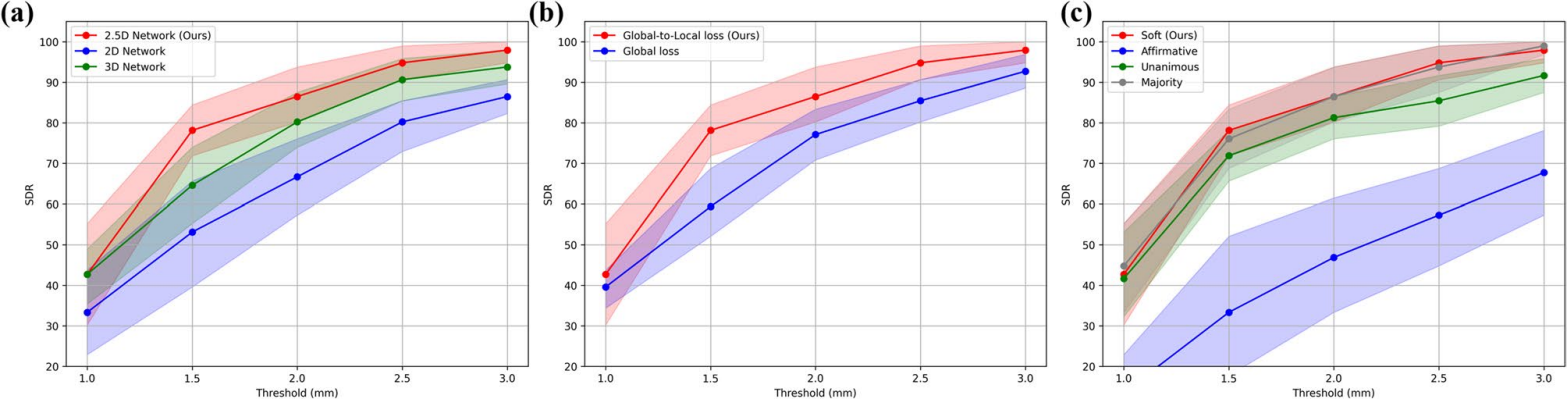
Success detection rate (SDR) curves. Shaded bands denote 95% confidence intervals estimated by bootstrap (B = 10,000). **a** Network dimensionality; 2D and 2.5D network: U-Net with EfficientNet-B3, and 3D network: V-Net. **b** Global-to-Local and Global loss on 2.5D network with EfficientNet-B3 (**c**) Network ensemble methods (Soft, Affirmative, Unanimous, Majority) on 2.5D network with EfficientNet-B3

The 2.5D network with EfficientNet-B3 demonstrated superior performance, achieving an MRE of 1.19 ± 0.65 *mm* and an SDR of 86.46% @ 2.0 *mm*, significantly surpassing both the 2D and 3D approaches (Table 1). Its SDR curve showed that it outperformed other best-performing 2D and 3D networks in all thresholds (Fig. 4). Prior 3D studies reported MREs ranging from 1.88 ± 1.10 *mm* to 7.61 ± 3.61 *mm* [56], indicating that our approach achieves competitive accuracy with recent literature. In terms of computational cost, it required 11.37 M parameters and 11.23G FLOPs, with 12.47 GB of peak memory, and measured a per-volume latency of 51.52 s. While its latency was longer than that of 2D networks, it remained substantially lower than the 3D. Therefore, the 2.5D approach provides a balanced option for accurate landmark localization with lower computational overhead, which is well-suited for deployment on entry-level cloud GPUs. Specifically, with 2.5D networks, most landmarks - except for Point.A and Porion - exhibited narrower error distributions and satisfied the clinically acceptable criteria (Fig. 3). Point.A is defined as the deepest concavity along the anterior maxillary profile rather than a discrete bony landmark, yielding flatter heatmaps and reduced sensitivity. Porion is located at the superior point of the external acoustic meatus. However, the thin cortical rim at the meatus roof yielded low-contrast, partial-volume–prone edges, so the superior point fluctuated across slices [56].

We assessed the impact of various backbones on landmark localization performance (Table 1). Among them, EfficientNet-B3 achieved the optimal trade-off between accuracy and computational efficiency. In both 2D and 2.5D dimensions, it consistently outperformed the others in terms of the MRE and SDR, while requiring significantly fewer computational loads. Notably, the transformer-based backbone MiT-B2 showed low accuracy, despite its higher computational load. Although transformers excel at capturing the global context using self-attention [56], landmark localization depends heavily on fine-grained local information, where even minor discrepancies can significantly degrade accuracy [61]. Conversely, CNNs has been known for their ability to extract detailed features [61], which makes them well-suited for landmark localization. Overall, our results demonstrated that EfficientNet-B3 was the most suitable backbone for balancing accuracy and computational cost in 3D landmark localization.

**Fig. 5:**
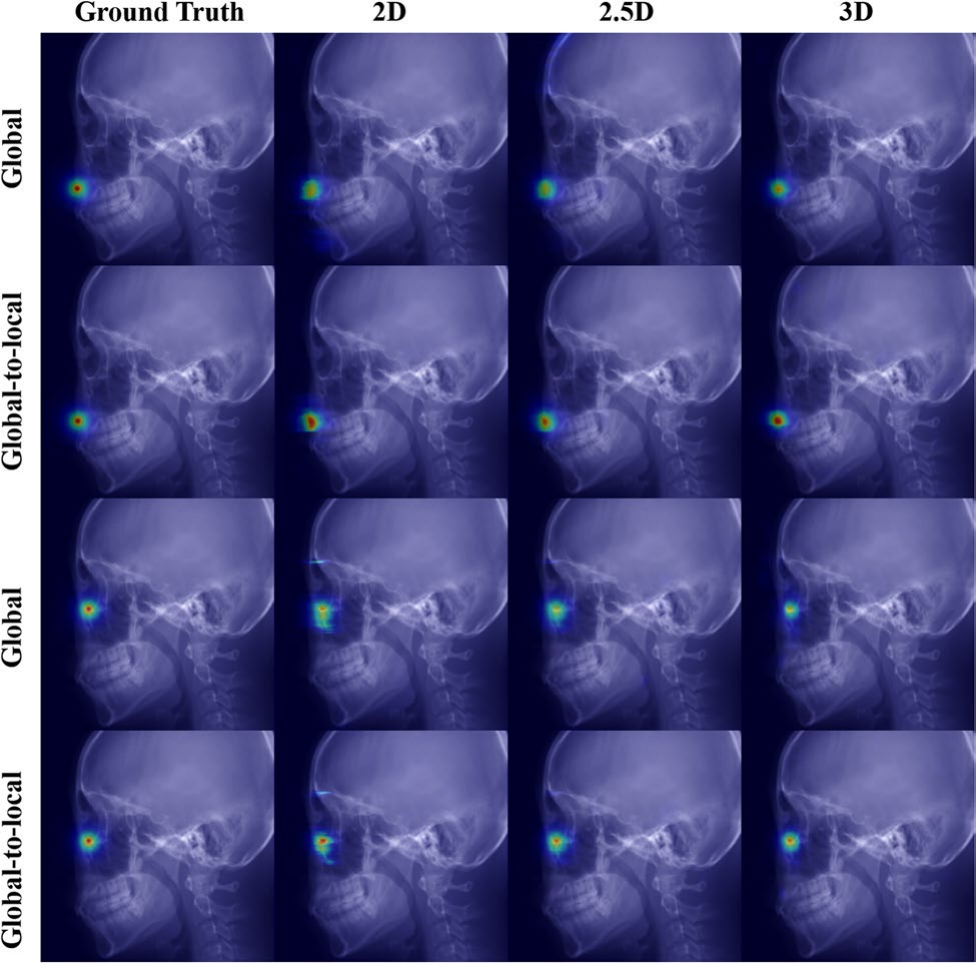
Heatmap comparison under global and global-to-local loss functions across network dimensions (2D, 2.5D, and 3D). The results for 2D and 2.5D networks are obtained from U-Net with EfficientNet-B3 backbone, and the 3D results are obtained from V-Net. Heatmaps using the global-to-local loss exhibit sharper, more concentrated activations and higher peak intensities at the landmark locations than those using global loss alone

The proposed global-to-local loss outperformed the conventional global loss, as summarized in Table 2. While the global loss supervises the entire heatmap, it is often dominated by the extensive background when the foreground occupies only a small fraction of pixels [47]. This background-foreground imbalance dilutes gradients around the true landmark and degrades performance. In contrast, the local term constrains optimization to a neighborhood around the landmark, strengthening gradients on fine-grained contextual cues. Figure 5 shows that heatmaps trained with the global-to-local loss exhibit sharper spatial distributions with higher peaks, whereas global-only training yields broader responses. However, we observed no significant difference (*p* = 0.998) between global-only and global-to-local losses for the 3D model. Patch-wise training intrinsically reduced foreground–background imbalance by sampling smaller 3D regions, which diminished the additional benefit of the local term. We also evaluated only local loss for training, but it generated too many false positives to localize the landmarks. Consequently, the combined objective balances background suppression (global) with focused refinement (local), thereby improving landmark localization.

**Fig. 6:**
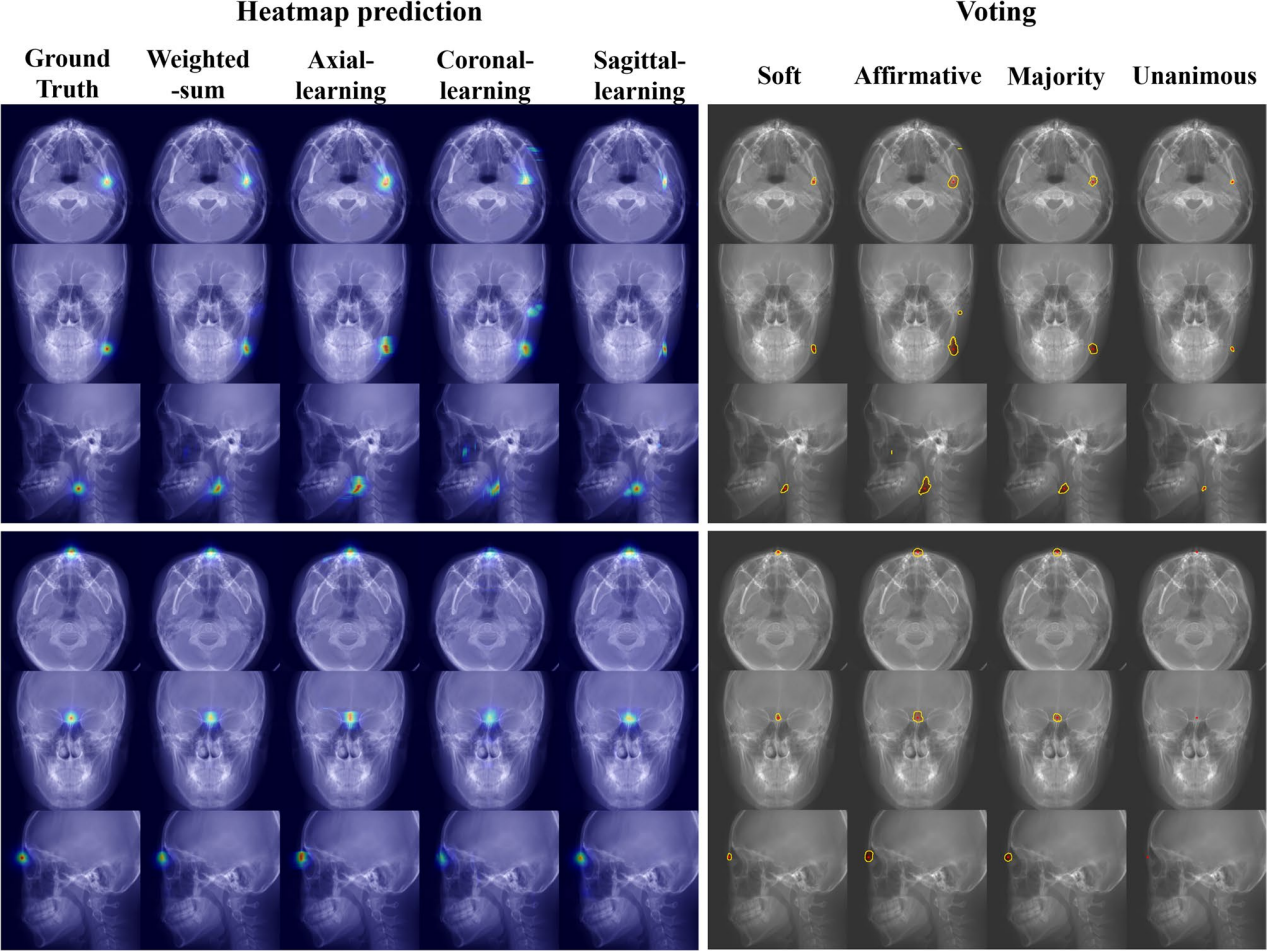
Comparison of landmark localization results using different ensemble voting methods in the 2.5D networks with an EfficientNet-B3 backbone. (Left) Three-dimensional stack of the heatmap predictions obtained from networks trained on axial, coronal, sagittal views, shown here as (from left to right) the ground-truth, the weighted sum of the three directional predictions, and the individual axial-only, coronal-only, and sagittal-only maps. (Right) Voting results using four different voting methods: soft and hard (affirmative, majority, and unanimous). Yellow contours indicate the selected voxels by each voting method, while the red dot represents the ground truth

In the network ensemble, soft voting showed the lowest MRE compared with hard voting methods (Table 3). Soft voting aggregated all directional-heatmap values before binarization, which preserved sub-threshold yet informative responses. However, hard voting first binarized each heatmap, which discarded sub-threshold informative responses. This difference made the soft voting more stable for landmark localization without misidentification. As illustrated in Fig. 6, the unanimous voting failed to detect the Naison landmark due to the weakened signal in the coronal heatmap. However, soft voting could localize the landmark by aggregating all directional-heatmap values. The affirmative voting reported the lowest performance, since its criteria was classified as a landmark if any of the three heatmaps exceeded a given threshold [43, 44]. Consequently, soft voting exhibited numerically favorable and stable performance, which could contribute to more reliable and accurate landmark detection. Among hard-voting schemes, majority voting was the most reasonable alternative. Although it was statistically different from soft voting, it yielded no misidentifications, and its SDR curve closely tracked that of soft voting in our test set (Fig. 4).

This study had limitations. Our dataset is limited to volumetric CT images from 40 patients. Furthermore, craniofacial morphology varies across age ranges and ethnic groups, whereas our dataset comprises a narrow age range and a single ethnic population. Therefore, our results should not be over-generalized beyond the studied cohort. Beyond dataset size and diversity, our study did not explicitly test stability to real-world factors such as image noise, missing slices, metal or motion artifacts, and non-standard head positioning. These issues are common in clinical CT and can reduce model accuracy. Although network ensembles help buffer small perturbations, a systematic assessment of reliability under degraded input was not performed. In future work, we will expand this dataset with multi-center data, and perform external validation to assess generalizability across sites and populations and clinical applicability.

## Conclusion

This study presents a comprehensive comparison of 2D, 2.5D, and 3D networks for three-dimensional cephalometric landmark localization, evaluating both localization accuracy and computational efficiency. Our results demonstrate that the 2.5D networks achieves the best trade-off performance, recording an MRE of 1.19 ± 0.65 *mm* and an SDR of 86.46% at 2.0 *mm*, while requiring the fewest computational resources. Additionally, we introduce a global-to-local loss function that balances foreground and background, and a soft voting that harmonized the continuous probabilistic results across views. These approaches improve the accuracy and robustness of 3D landmark localization. Despite these promising outcomes, our study is limited to a single-center dataset of 40 patients without artifacts, which may limit the generalizability. Future work will extend the evaluation to multicenter datasets, assessing performance and stability to distortion in diverse patient populations. We also plan to apply the 2.5D paradigm and loss formulations to other anatomical landmark detection tasks.

## Supplementary Information

Supplementary Material 1.

## Data Availability

The datasets generated and/or analyzed during the current study are not publicly available due to the restriction by the Institutional Review Board (IRB) of Seoul National University Dental Hospital in order to protect patient’s privacy but are available from the corresponding author on reasonable request.

## Abbreviations

CT: Computed tomography
ROI: Region of interest
HU: Hounsfield units
IRB: Institutional review board
ICC: Intraclass correlation coefficient
CNN: Convolutional neural network
CoM: Center of mass
MSE: Mean squared error
GPU: Graphic processing unit
MRE: Mean radial error
SDR: Success detection rate
FLOPs: Floating-point operations
VRAM: Video random access memory
BCa: Bias-corrected and accelerated
