# Supplementary material for: Comparison of 2D, 2.5D, and 3D landmark localization networks for 3D cephalometry in CT images
Source: BMC Oral Health. 2025 Nov 26;25:1843. doi: 10.1186/s12903-025-07189-3 (PMC12659582; doi:10.1186/s12903-025-07189-3)
Supplement: Supplementary file 1 — Supplementary Material 1. [file 12903_2025_7189_MOESM1_ESM.docx]

- **Supplementary Table 1. Quantitative results of a grid search over soft-voting fusion weights across direction (Axial/Coronal/Sagittal). For each weight set, MRE is reported as mean with 95% CI, median, and IQR (25th–75th percentile). The validation split was used to select the best weights, which were then frozen and evaluated on the test split.**

| **Dataset** | **Weights** | | |  | | **MRE ↓*(mm)*** | | | |
| --- | --- | --- | --- | --- | --- | --- | --- | --- | --- |
|  | **Axial** | **Coronal** | **Sagittal** | |  | | **Mean, 95% CI** | **Median** | **IQR** |
| **Validation** | 0.33 | 0.33 | 0.33 | |  | | 1.22, [1.10, 1.36] | 0.93 | [0.60, 1.52] |
|  | 0.50 | 0.25 | 0.25 | |  | | 1.30, [1.20, 1.47] | 0.98 | [0.68, 1.79] |
|  | 0.25 | 0.50 | 0.25 | |  | | 1.18, [1.09, 1.36] | 0.95 | [0.58, 1.48] |
|  | 0.25 | 0.25 | 0.50 | |  | | 1.19, [1.08, 1.33] | 0.97 | [0.55, 1.59] |
| **Test** | 0.33 | 0.33 | 0.33 | |  | | 1.20, [1.08, 1.35] | 1.04 | [0.78, 1.46] |
|  | 0.50 | 0.25 | 0.25 | |  | | 1.29, [1.17, 1.45] | 1.20 | [0.86, 1.52] |
|  | 0.25 | 0.50 | 0.25 | |  | | 1.19, [1.08, 1.33] | 1.06 | [0.74, 1.34] |
|  | 0.25 | 0.25 | 0.50 | |  | | 1.20, [1.07, 1.35] | 1.04 | [0.73, 1.47] |

- **Supplementary Table 2. The MREs of landmark localization performance across different network dimensions (2D, 2.5D, and 3D) and backbones. Values are reported as mean (95% CI), median and IQR. p-values are form Wilcoxon signed-rank test at 0.05 of significance level between each variant and the 2.5D network with EfficientNet-B3.**

| **Network dimensions** | **Backbone** | **MRE ↓ *(mm)*** | | | |  |
| --- | --- | --- | --- | --- | --- | --- |
|  |  | **Mean, 95% CI** | **Median** | **IQR** | ***p*** | |
| **2 D** | **ResNet18** | 1.80, [1.59, 208] | 1.65 | [0.91, 2.23] | $<$0.001 | |
|  | **DenseNet169** | 1.77, [1.58, 2.00] | 1.54 | [0.92, 2.48] | $<$0.001 | |
|  | **MiT-B2** | 2.24, [1.92, 2.66] | 1.65 | [1.12, 2.88] | $<$0.001 | |
|  | **EfficientNet-B3** | $1.76$, [1.54, 203] | 1.43 | [0.90, 2.31] | $<$0.001 | |
| **2.5D** | **ResNet18** | 1.38, [1.23, 1.53] | 1.18 | [0.88, 1.68] | $<$0.001 | |
|  | **DenseNet169** | 1.26, [1.13, 1.43] | 1.09 | [0.80, 1.45] | 0.089 | |
|  | **MiT-B2** | 1.39 [1.23, 1.58] | 1.15 | [0.77, 1.92] | 0.024 | |
|  | **EfficientNet-B3** | 1.19, [1.08, 1.33] | 1.06 | [0.74, 1.34] | - | |
| **3 D** | **U-Net** | 1.59, [1.41, 1.80] | 1.35 | [0.90, 1.96] | $<$0.001 | |
|  | **V-Net** | 1.38, [1.22, 1.60] | 1.20 | [0.73, 1.78] | 0.025 | |

- **Supplement Table 3. The localization accuracy for landmarks (Sella, Nasion, Orbitale, ANS, Point.A) using 2D and 2.5D U-Net with Efficient-B3 backbone, and 3D V-Net. Values are reported as mean (95% CI), median and IQR. p-values are form Wilcoxon signed-rank test at 0.05 of significance level between each variant and the 2.5D network.**

| **Landmark** | **Network dimensions** | **MRE ↓*(mm)*** | | | |
| --- | --- | --- | --- | --- | --- |
|  |  | **Mean, 95% CI** | **Median** | **IQR** | ***p*** |
| **Se** | 2D | 1.12, [0.79, 1.48] | 0.95 | [0.78, 1.66] | <0.001 |
|  | 2.5D | 0.63, [0.52, 0.79] | 0.59 | [0.56, 0.68] | - |
|  | 3D | 0.89, [0.67, 1.14] | 0.79 | [0.54, 1.22] | 0.055 |
| **Na** | 2D | 3.59, [2.86, 4.72] | 3.04 | [2.73, 4.13] | <0.001 |
|  | 2.5D | 1.02, [0.59, 1.59] | 0.96 | [0.59, 1.35] | - |
|  | 3D | 1.28, [0.82, 1.71] | 1.40 | [0.61, 1.83] | 0.383 |
| **Or(L)** | 2D | 1.01, [0.59, 1.57] | 0.91 | [0.49, 1.28] | 0.74 |
|  | 2.5D | 1.07, [0.92, 1.26] | 1.03 | [0.93, 1.23] | - |
|  | 3D | 0.91, [0.61, 1.23] | 0.77 | [0.59, 1.32] | 0.195 |
| **Or(R)** | 2D | 1.52, [1.09, 1.98] | 1.43 | [0.88, 2.13] | 0.380 |
|  | 2.5D | 1.23, [0.90, 1.62] | 1.23 | [0.98, 1.42] | - |
|  | 3D | 1.33, [0.94, 1.82] | 1.23 | [0.89, 1.69] | 0.641 |
| **ANS** | 2D | 1.12, [0.90, 1.32] | 1.16 | [0.87, 1.41] | 0.38 |
|  | 2.5D | 0.95, [0.82, 1.06] | 0.99 | [0.80, 1.08] | - |
|  | 3D | 1.20, [0.91, 1.48] | 1.20 | [0.93, 1.55] | 0.383 |
| **Point.A** | 2D | 1.60, [0.90, 2.63] | 1.02 | [0.73, 2.19] | 0.94 |
|  | 2.5D | 1.63, [1.07, 2.29] | 1.31 | [0.88, 2.50] | - |
|  | 3D | 1.77, [1.28, 2.43] | 1.77 | [0.96, 2.23] | 0.547 |

- **Supplement Table 4. The localization accuracy for landmarks (Menton, Pogonion, Gonion, Porion) using 2D and 2.5D U-Net with Efficient-B3 backbone, and 3D V-Net. Values are reported as mean (95% CI), median and IQR. p-values are form Wilcoxon signed-rank test at 0.05 of significance level between each variant and the 2.5D network.**

| **Landmark** | **Network dimensions** | **MRE ↓*(mm)*** | | | |
| --- | --- | --- | --- | --- | --- |
|  |  | **Mean, 95% CI** | **Median** | **IQR** | ***p*** |
| **Me** | 2D | 1.47, [1.19, 1.76] | 1.42 | [1.14, 1.79] | 0.02 |
|  | 2.5D | 1.10, [0.91, 1.31] | 1.18 | [0.80, 1.26] | - |
|  | 3D | 0.91, [0.68, 1.21] | 0.93 | [0.68, 1.11] | 0.250 |
| **Pg** | 2D | 1.67, [1.18, 2.46] | 1.39 | [1.12, 2.06] | 0.31 |
|  | 2.5D | 1.19, [0.79, 1.64] | 1.06 | [0.66, 1.73] | - |
|  | 3D | 1.26, [0.98, 1.54] | 1.26 | [1.03, 1.54] | 0.742 |
| **Go(L)** | 2D | 2.27, [1.40, 3.14] | 2.44 | [1.38, 2.84] | 0.04 |
|  | 2.5D | 1.08, [0.93, 1.20] | 1.07 | [1.00, 1.29] | - |
|  | 3D | 1.04, [0.73, 1.46] | 0.92 | [0.76, 1.28] | 0.641 |
| **Go(R)** | 2D | 2.26, [1.42, 3.76] | 2.43 | [0.86, 2.52] | 0.15 |
|  | 2.5D | 1.33, [1.03, 1.68] | 1.31 | [1.03, 1.61] | - |
|  | 3D | 1.64, [0.65, 3.32] | 0.61 | [0.36, 2.33] | 0.945 |
| **Po(L)** | 2D | 1.81, [1.25, 3.02] | 1.59 | [0.90, 2.08] | 0.74 |
|  | 2.5D | 1.74, [1.09, 2.60] | 1.21 | [0.77, 2.72] | - |
|  | 3D | 2.31, [1.75, 3.42] | 2.31 | [1.47, 2.75] | 0.023 |
| **Po(R)** | 2D | 1.65, [1.21, 2.50] | 1.44 | [1.11, 1.89] | 0.25 |
|  | 2.5D | 1.33, [0.99, 1.86] | 1.04 | [1.02, 1.46] | - |
|  | 3D | 1.99, [1.30, 2.76] | 2.11 | [1.38, 2.36] | 0.055 |
